# Early cancer detection in primary care in Ireland: a protocol for a research prioritisation exercise

**DOI:** 10.12688/hrbopenres.13749.1

**Published:** 2023-09-13

**Authors:** Benjamin M. Jacob, Laura O'Connor, Barbara Clyne, Heather Burns, Surour Alneyadi, Primary Care PPI Group (HRB Primary Care Clinical Trials Network), Richard D Neal, Patrick Redmond

**Affiliations:** 1 Department of General Practice, RCSI University of Medicine and Health Sciences, Dublin, Ireland; 2 HRB Primary Care Clinical Trials Network Ireland, University of Galway, Galway, Ireland; 3 Department of Public Health & Epidemiology, RCSI University of Medicine and Health Sciences, Dublin, Ireland; 4 National Cancer Control Programme, Health Service Executive, Dublin, Ireland; 5 Department of Health and Community Sciences, Faculty of Health and Life Sciences, University of Exeter, Exeter, UK

**Keywords:** Research priority setting; Patient and Public Involvement; Primary care; General Practice; Early Detection of Cancer; Ireland

## Abstract

**Background:** Cancer is a significant cause of morbidity, mortality, and economic loss in Ireland. It is important that cancer research funding is directed in accordance with the values of a wide variety of stakeholders, so as to ultimately deliver tangible benefits to cancer patients. The aim of this study is to achieve consensus among key stakeholders (including patients, caregivers, healthcare professionals, policymakers and academics) regarding research priorities in the area of early detection of cancer in primary care in Ireland.

**Methods**: A research prioritisation exercise adapted from the James Lind Alliance (JLA) consensus framework will be used to enable all key stakeholders to identify and prioritise research questions. This involves the following stages: (1) setting up a steering group and priority setting partnership, (2) gathering potential research questions via an online survey, (3) processing, categorising, and summarising these research questions, (4) identifying the unanswered research questions, (5) determining the top 10 research priorities via a consensus workshop.

**Results**: The following outcomes will be reported: (1) a “Top 10” list of the most important research questions in early cancer detection in primary care; (2) a list of unanswered research questions which ranked outside of the Top 10; (3) a list of research questions which were proposed but considered to be already answered by a panel of academics working in the field.

**Conclusions**: The co-production of consensus derived research questions in early cancer detection will provide a platform for both funders and researchers to concentrate on the most significant issues to stakeholders, especially patients and their doctors.

## Introduction

Cancer imposes a significant healthcare burden on Ireland with an average of 35,825 invasive cancers diagnosed annually between 201–2020 ^1^. Cancers diagnosed at an advanced stage are associated with poor prognosis ^2^. Early detection—i.e., finding tumours before they spread—offers the greatest potential for cure, in addition to reducing morbidity and the cost of treatment ^2^.

In addition to cancer accounting for 5% of all health expenditure in Ireland ^3^, cancer research is a major destination for health research funds ^4^. Research funding is not allocated in proportion to the burden of disease nor its potential impact on population health ^5– 7^. It is known to be biased towards existing areas of research, influenced by the capacity and ‘track record’ of successful researchers ^5, 6^. “Early cancer detection, diagnosis and prognosis” research receives 16% of all cancer research funding in the UK, with “Biology” and “Treatment” sectors each receiving double this amount ^8^. Primary care research, the key player in early cancer detection ^9– 11^, is known to be underfunded relative to laboratory and secondary care research ^12– 16^.

Research prioritisation exercises aim to address this mismatch and highlight (to researchers, funders, and industry) questions of most interest and relevance to key stakeholders ^17^. The process involves identifying research priorities among a diverse stakeholder group, mapping corresponding gaps in the evidence, and ranking them by importance in accordance with the priorities of the stakeholders ^18^. There is evidence that this leads to efficient allocation of funding resources and decreases research waste ^19– 21^.

The James Lind Alliance (JLA) is a British initiative, established in 2004, which seeks to “bring patient, carers and clinicians together to identify and prioritise the unanswered questions they want health research to address” in groups known as “Priority Setting Partnerships” (PSPs) ^18, 22, 23^. In the UK, in 2019, the “Detecting Cancer Early PSP” produced a “Top 10” list of research questions in this area: five related to testing for cancer, three related to using patient data more effectively, one related to identifying social risk factors for late diagnosis, and one related to tumour pathology ^24, 25^.

### Rationale, aims and objectives

However, given the substantial differences in primary healthcare contexts between the UK and Ireland ^26^, it is important to develop a consensus around research priorities in an Irish setting. The aim of this study is to achieve consensus regarding the shared research priorities of patients, caregivers, healthcare professionals and academics in relation to early detection of cancer in primary care in Ireland.

## Methods

**Figure 1.**
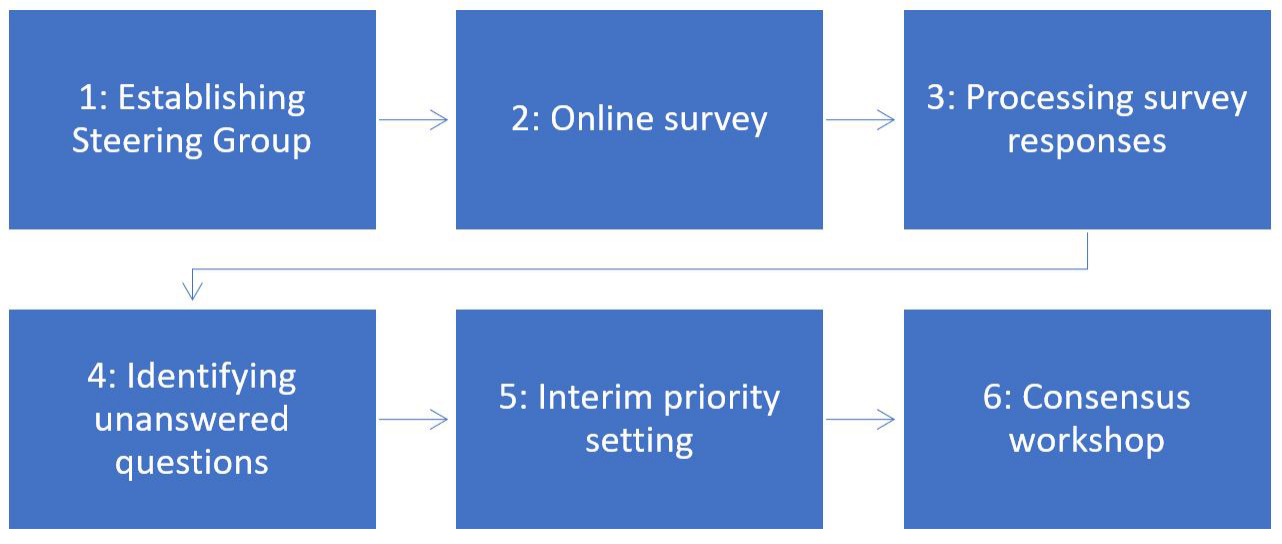
Stages of the research prioritisation exercise.

A research prioritisation exercise will be conducted, informed by the James Lind Alliance framework, comprising an online survey, literature review, interim priority setting, and in-person consensus workshop. We will utilise the standardised reporting guideline for priority setting of health research (REPRISE) ^27^. The six stages of the prioritisation exercise are illustrated in Figure 1.

### Beneficiary

The ultimate beneficiary of the research priority setting will be the Irish public who will gain from any resulting improvements to cancer care which permit earlier detection, thereby allowing more effective treatment and better outcomes ^28^.

### Scope

The scope of this research prioritisation exercise is limited to the early detection of symptomatic adult cancer in primary care in Ireland. In the cancer continuum, “early detection” refers to the first diagnosis of cancer. Ireland is a sovereign state often referred to as the “Republic of Ireland”, and should not be confused with “Northern Ireland” which is one of four “nations” which make up the United Kingdom.

### Healthcare context

The Irish primary healthcare system is characterised by mixed public/private funding — GP visits are free for only 40% of residents (roughly the poorest one-third of society, under 6s, over 70s and those with eligible medical conditions) with either a “medical card” or a “GP visit card” ^26, 29^. The remainder pay €53 on average to visit the GP ^30^. Population-based cancer screening programmes are offered for breast, cervical and colorectal cancer ^26^. Universal free access to cancer treatment is delivered by the Health Service Executive ^31, 32^, however, private healthcare also plays a role in the provision of cancer diagnostics and cancer treatment for those who can afford it ^26^.

### 1: PSP Steering group

In the JLA framework, the entire research prioritisation project—known as a “Priority Setting Partnership” (PSP)—is designed and conducted by a “PSP Steering Group”, a stakeholder group representing patients, carers and clinicians, whose meetings are chaired by a JLA adviser. Due to resource constraints, our steering group will be convened via a parallel initiative to establish a permanent stakeholder group to support primary care cancer research in Ireland [cite HRB Open reference]. While the primary care cancer research stakeholder group will have a broad membership, the research prioritisation steering group will comprise a subgroup of PPI and clinician stakeholders, and members of the research team. Reimbursement for PPI contributors will be funded through a grant from the Irish Cancer Society. This is consistent with the JLA principles that there is a “balanced inclusion of patient, carer and clinician interests” with “exclusion of non-clinician researchers from voting” and “exclusion of significant competing [i.e., corporate] interests” ^23^. Additionally, co-authors LOC and the Primary Care PPI group have given input to this protocol based on their experience of a JLA supported PSP, currently being undertaken by the HRB Primary Care Clinical Trials Network ^30^.

The main roles of the steering group will be (1) providing guidance on the selection and wording of the questions in the online survey, (2) providing guidance on its dissemination, (3) offering oversight of the processing of responses in the interim priority-setting stage, and (4) providing guidance on the running of the consensus workshop.

### 2: Online survey

Firstly, an online survey will be conducted to identify aspects of early detection of cancer in primary care research perceived as important– Table 1 ^33, 34^.

**Table 1.** Online questionnaire.

Section 1 (Experience of cancer)	
[1] What is your main experience of cancer?	Cancer patient / Carer to a cancer patient / Other personal experience of cancer / Doctor / Allied health professional / Researcher / Policymaker / Other professional experience of cancer
Section 2 (Research ideas)	
[2] Think about someone you know who has experienced cancer (perhaps it’s you). Reflect on their journey from the beginning until they received a diagnosis. Do you have any ideas about how we could detect cancer earlier?	[Free-text answer]
[3] Think about the role played by “primary care professionals” (GPs, GP nurses, Public Health Nurses, Pharmacists, Dentists and any healthcare workers in your *community*). What research should we be doing to help these professionals detect cancer earlier?	[Free-text answer]
Section 3 (Basic information)	
[4] Age?	Less than 20 / 20-29 / 30-39 / 40-49 / 50-59 / 60-69 / 70-79 / 80+
[5] Gender? [6] Do you live in the Republic of Ireland?	Male / Female / Other Yes / No

***Recruitment.*** Recruitment will be targeted at patients, carers, clinicians, policymakers, and academics based primarily in Ireland that self-identify as being interested in the topic area. The survey will be sent via established mailing lists, professional networks, collaborator networks (e.g., National Cancer Control Programme, Irish Cancer Society, Marie Keating Foundation etc.), and social media channels. Advertising will include a video explaining the content and aims of the research prioritisation exercise. The survey period will be for 6 weeks, with an extension if required to achieve a minimum of 25–30 unique research questions, in keeping with JLA advice regarding the typical number included in the final prioritisation workshop ^23, 35^.

***Content.*** The survey, developed with PPI input, will collect information, via a mixture of closed and open questions on the participant’s experience of cancer and their proposed research ideas ( Table 1). Questions about participant demographics are placed at the end, to confirm broad circulation of the survey.

### 3: Processing survey responses

The survey results will be processed via the following steps: (1) removal of unintelligible or inappropriate responses; (2) paraphrasing responses into a question format where needed, (3) merging of duplicate questions into a single question, and (4) removal of research questions outside the scope. This will yield a list of unique questions. Steering group members will review the list of questions to ensure they are a true reflection of initial submissions and are worded clearly.

Survey responses related to paediatric cancers, other areas of the cancer continuum (e.g., screening, treatment or survivorship), or cancer control outside of a community setting will not automatically be deemed out-of-scope. Rather, the authors will attempt to narrow the scope of the proposed research question, and only if this is unsuccessful will the survey response be excluded. Exclusions based on scope will be reported in an appendix.

### 4: Identifying unanswered questions

The research questions will then be checked against the existing evidence base. To expedite the process, the list will be sent to academic and clinical colleagues working in early cancer detection. If a systematic review or meta-analysis definitively answers a proposed research question, and is applicable to an Irish context, this question will be removed, given agreement of the steering group. Where uncertainty remains (as to existence of an adequate study or clinical guideline), a literature review will be conducted. Where the steering group cannot agree on the existence of research which adequately answers a research question, that question will be included. Methodology-specific quality assessment tools, such as those produced by the National Institute for Health ^36^, will be used if necessary.

### 5: Interim priority setting

It is likely that the resulting longlist of unanswered research questions remains too long to allow feasible discussion by the workshop participants (in the research prioritisation literature, 20–30 questions is typical) ^23, 35^. Hence, JLA PSPs often utilise a process called “interim priority setting” whereby the longlist of research questions is subject to a prioritisation exercise, remotely, prior to the workshop ^23, 35^. In our case, we will ask the already-identified workshop participants (rather than the wider public due to time constraints) to rank their top 20 questions ^23, 35^. The questions featuring the lowest number of votes will be excluded in order to produce a shortlist of approximately 20 questions.

### 6: Consensus workshop

Finally, we will convene a workshop, the purpose of which is to take the 20-item shortlist produced by the interim priority setting stage and produce, via consensus exercises, a ranked “Top 10” of research priority questions for detecting cancer early.

***Recruitment.*** Recruitment will begin during the survey phase, when respondents from the eligible stakeholder groups (patient, carer, clinician, academic, policymaker) will be invited to participate in a consensus workshop. Targeted invitations to attend the workshop will also be circulated by the same channels utilised when advertising the survey (mailing lists, professional networks, and collaborator networks). They will be provided with a patient information leaflet and a video explaining what the workshop entails. Furthermore, a short video will also be produced to promote the event and shared on social media to raise awareness.

A minimum of five stakeholders will be recruited in each of three broad stakeholder groups: (1) patients and carers; (2) clinicians; (3) academics and policymakers. Enrolment will be limited to ensure that no one of these groups consists of more than 50% of the total participants. Workshop participants must be: English-speaking, with personal or professional experience of cancer care in the Irish healthcare service, 18 years or older, and able to provide informed consent to participate. Where possible, in the scenario where the workshop is oversubscribed, in addition to achieving balance between stakeholder groups, we will seek to include a diversity of age, gender, and cancer type. Participants will be reimbursed for travel expenses.

***Format.*** At the workshop, which will be coordinated by the research team, the participants will be divided into small groups (5–10) with at least 1–2 members from each stakeholder group, and will engage in multiple rounds of small group discussions and ranking exercises in accordance the procedures outlined in the JLA Guidebook ^23^. The workshop will culminate with a whole group review with the intention of arriving at a finalised Top 10 list.

The in-person workshop encourages the participants to voice their own beliefs regarding the relative importance of various research questions. The iterative nature of the process will allow the participants to reflect upon and integrate the opinions of others (especially those from different stakeholder groups) such that the group converges on a consensus position and a finalised Top 10 list.

## Results

We will report (1) a “Top 10” list of the most important research questions in early cancer detection in primary care, as determined by the process, and (2) the number of research questions or topics considered, highlighting the numbers removed at each stage of the process.

We will describe the selection, structure and characteristics of the steering group responsible for “initiating, developing, and guiding the process of priority setting”, in accordance with REPRISE guidance. In addition, we will describe the characteristics of stakeholders involved in the research prioritisation workshop: their stakeholder group designation, their demographic characteristics, as well as their areas of interest, expertise, and institutional affiliations where appropriate.

Finally, we will also detail, in an appendix, the following: (1) a list of unanswered research questions which ranked outside of the Top 10 or were excluded by the interim priority setting; (2) a list of research questions which were proposed but considered to be already answered by a panel of academics working in the field; (3) a list of research questions which were proposed but considered to be out-of-scope by the steering group.

## Dissemination

In addition to a peer-reviewed open access publication and academic conference presentations, we will also disseminate the findings to a broad range of professional networks with relevance to early cancer detection or primary care research. Moreover, we intend to promote the top ten research priorities at a public event focusing on cancer control in primary care.

The steering group will identify appropriate audiences to engage when sharing the results, such as researchers, funders, and patient and clinical communities, highlighting opportunities to contribute evidence to answer the newly identified research priorities, and opportunities to work with policymakers and clinicians to ensure findings are translated into practice.

## Study status

At the time of submission, planning of the online survey was underway, but the survey had not yet been released to the public.

## Conclusion and implications

By this research prioritisation exercise, we will produce a consensus statement on the most important research questions pertaining to early cancer detection in primary care in Ireland, based meaningfully and transparently on the values and experience of patients, carers and clinicians. This will be used to encourage funders, researchers, and industry to focus their attention and resources on issues of importance to these groups.

## Ethics

Ethical approval granted by the RCSI REC (Ethics Review Code: 202210025).

## Data Availability

No data are associated with this article.
