# Supplementary material for: Early detection of symptomatic cancer in primary care in Ireland: a protocol for a research prioritisation exercise
Source: HRB Open Res. 2025 Nov 11;6:52. Originally published 2023 Sep 13. [Version 2] doi: 10.12688/hrbopenres.13749.2 (PMC12612614; doi:10.12688/hrbopenres.13749.2)
Supplement: Peer review history

# Reviewer report: Reviewer response for version 2

**DOI:** 10.21956/hrbopenres.15726.r51372

**Published:** 2025-11-12

**Authors:** Yasemin Hirst

**Affiliations:** 1 Applied Health Research Hub,, University of Central Lancashire, Preston, England, UK

Thank you for the revised protocol. Happy with how the authors addressed all my queries.

Is the study design appropriate for the research question?

Yes

Is the rationale for, and objectives of, the study clearly described?

Yes

Are sufficient details of the methods provided to allow replication by others?

Yes

Are the datasets clearly presented in a useable and accessible format?

Not applicable

Reviewer Expertise:

Cancer prevention and early diagnosis, population health, mixed methods research

I confirm that I have read this submission and believe that I have an appropriate level of expertise to confirm that it is of an acceptable scientific standard.

# Reviewer report: Reviewer response for version 1

**DOI:** 10.21956/hrbopenres.15040.r37176

**Published:** 2024-09-19

**Authors:** Yasemin Hirst

**Affiliations:** 1 Applied Health Research Hub,, University of Central Lancashire, Preston, England, UK

Many thanks for the opportunity to review this protocol paper that aims to carry out a research prioritisation exercise following the JLA framework in Ireland. 

 It is a well-written protocol that provides a broad detail about the healthcare context in Ireland and cancer outcomes. 

 It would be great, if data exists, to include some details about the inequalities in cancer diagnosis in Ireland specific to routes to diagnosis and primary care. If this data is not available, perhaps it is important to highlight why it is important to investigate symptomatic primary care pathway. I think what is missing here is a summary of what we know vs what we don't know to build the need for the priority setting. 

 The paper also highlights the differences between the UK and Ireland healthcare system, are there differences for suspected cancer referral pathways too? 

 Methodologically, my main query relates to the inclusion criteria of respondents and in fact exclusion of those who are often under-represented in health research.I would encourage the authors to either justify the exclusion or ensure that they have representation from vulnerable communities e.g. d/Deaf, learning disabilities, physical disabilities, ethnic minorities, refugees, people with mental illness etc. Inclusion of these populations would be important to challenge whether the priorities are not widening inequalities because they do not capture inequities in healthcare. Capturing a detailed sociodemographic data throughout the project would help with the generalisability as well as minimise bias. 

 Q2 in the online questionnaire, I believe this survey was developed with the PPI input, however I have some concerns about the wording and potential distress it may cause for some people especially if the cancers may have been asymptomatic for a long period of time and resulted a late stage diagnosis. Perhaps it would be better to ask people what could be done to improve cancer diagnosis after an individual presents in primary care with symptoms that could be a sign of cancer. This gives better focus on the primary care interval and removes any guilt or concerns people might have about the patient interval before the diagnosis which might also relate to delays in presentation. The main reason I'm highlighting this because people are asked to reflect on their journey. 

 My personal opinion, the word "detect" indicates that the individual has been exposed to a diagnostic tool or an investigation. The words early detection and diagnosis are used interchangeably in the context of cancer research and new/media, I would encourage the authors to define these carefully and follow expert consensus. I do not agree with the statement re early detection is the first diagnosis of cancer. It would be good to have a reference to their statement and it might be good to define it as part of their consensus.  Cancer Research UK might have a well-structured definition. 

 Minor amendment, PIS is referred to as patient information sheet, this should be revised as participant information sheet.

Is the study design appropriate for the research question?

Yes

Is the rationale for, and objectives of, the study clearly described?

Yes

Are sufficient details of the methods provided to allow replication by others?

Yes

Are the datasets clearly presented in a useable and accessible format?

Not applicable

Reviewer Expertise:

Cancer prevention and early diagnosis, population health, mixed methods research

I confirm that I have read this submission and believe that I have an appropriate level of expertise to confirm that it is of an acceptable scientific standard, however I have significant reservations, as outlined above.

# Reviewer report: Reviewer response for version 1

**DOI:** 10.21956/hrbopenres.15040.r37439

**Published:** 2023-12-29

**Authors:** Xiaoyu Zong

**Affiliations:** 1 School of Medicine in St. Louis, Washington University, Seatle, Washington, USA

Jacob et al. aimed to reach a consensus on the prioritization of research regarding cancer detection in primary care with the JLA framework. The author gathered information from key stakeholders, and the protocol was well-structured. By filling the gaps in cancer detection research, the protocol would provide some ideas for the relevant intervention conducted in primary care settings afterward in Ireland.

 Some primary concerns:

 Rationale - The rationale focused on cancer detection in primary care was underfunded. The author used the situation in the UK as an example and referenced several instances in other countries. However, the benefits, such as the effectiveness and feasibility of studying cancer detection in primary care, and patient outcomes, could be further elaborated.

 Scope - Why does the scope emphasize symptomatic adult cancer in primary care? Patients are more likely to seek treatment from primary care when they experience symptoms rather than for preventative purposes.

 Online survey - What tools are you planning to use to process the Online questionnaire Section 2 open-ended questions?

Is the study design appropriate for the research question?

Yes

Is the rationale for, and objectives of, the study clearly described?

Yes

Are sufficient details of the methods provided to allow replication by others?

Partly

Are the datasets clearly presented in a useable and accessible format?

Not applicable

Reviewer Expertise:

Cancer risk, cancer survivor, cancer early detection

I confirm that I have read this submission and believe that I have an appropriate level of expertise to confirm that it is of an acceptable scientific standard, however I have significant reservations, as outlined above.

# Reviewer report: Reviewer response for version 1

**DOI:** 10.21956/hrbopenres.15040.r36808

**Published:** 2023-11-09

**Authors:** Kevin Monahan

**Affiliations:** 1 St Mark’s Hospital, Imperial College London, London, England, UK

This is a well designed study protocol which will provide a useful strategic perspective on the role of early detection of cancer in the Irish primary care setting. I have a couple of minor queries:

1. Why select 'early detection' and not also 'prevention' of cancer?
2. Will clinicians and academics be drawn from primary care and other care providers also? It may be helpful to understand a little more about professional representation and how it will be identified and appropriate i.e. what is meant by 'professional networks'?

Is the study design appropriate for the research question?

Yes

Is the rationale for, and objectives of, the study clearly described?

Yes

Are sufficient details of the methods provided to allow replication by others?

Yes

Are the datasets clearly presented in a useable and accessible format?

Yes

Reviewer Expertise:

Early detection and prevention of cancer

I confirm that I have read this submission and believe that I have an appropriate level of expertise to confirm that it is of an acceptable scientific standard, however I have significant reservations, as outlined above.
